# Contact with child protection services during pregnancy: a cross-sectional study using the eLIXIR Born in South London, UK maternity-child data linkage

**DOI:** 10.1186/s12884-025-08197-5

**Published:** 2025-10-16

**Authors:** Kaat De Backer, Paul Seed, Sam Burton, Elsa Montgomery, Jane Sandall, eLIXIR-Born in South London Partnership, Abigail Easter

**Affiliations:** 1 Department of Women and Children’s Health, Faculty of Life Sciences and Medicine, King’s College London, London, UK; 2 School of Psychology, Liverpool John Moores University, Liverpool, UK; 3 Division of Methodologies, Florence Nightingale Faculty of Nursing, Midwifery and Palliative Care, King’s College London, London, UK

**Keywords:** Maternity services, Child protection, Safeguarding

## Abstract

**Background:**

In the last decade, rates of children with child protection agency involvement have increased in many high-income countries, including the UK. Disparities in both maternal and child health outcomes as well as child welfare referrals have been widely evidenced, yet no previous research has investigated contact with child protection agencies in the UK (Children’s Social Care, CSC) during pregnancy using linked maternity and mental health records. The aim of this study was to investigate characteristics of pregnant women when child protection agencies are involved and investigate what risk factors are associated with child protection agency contact during pregnancy.

**Methods:**

We conducted a retrospective cross-sectional study using linked electronic health records from maternity, neonatal, and mental health services in South London (eLIXIR-BiSL cohort). A cohort of singleton pregnancy records was created (October 2018 – April 2023). We used descriptive statistics to investigate sociodemographic and clinical characteristics, and binomial regression to explore risk factors and characteristics associated with CSC contact during pregnancy.

**Results:**

A cohort of 36,322 singleton pregnancy records was studied, with CSC contact identified in 2,206 records (6%). CSC contact was most frequently observed among Black and multiparous women, and those living in poorer socio-economic circumstances. Those with CSC contact more often had recorded medical, obstetric and psychiatric comorbidities compared to those without CSC contact. When investigating referral indications associated with CSC contact, we found 1,733 pregnancy records with risk factors indicating referral concordant with local guidance yet without any CSC contact. In contrast, in 913 pregnancies CSC contact occurred without any prescribed referral indication being identified. In this group, CSC contact was more frequently associated with Black or mixed ethnicity, social deprivation, maternal unemployment, single motherhood, maternal age under 25 years, previous domestic abuse disclosures, and previous mental health or CSC contact.

**Conclusions:**

In this UK population cohort, socio-economic and ethnic disparities were observed between those in contact with child protection agencies during pregnancy and those without. This continued to be observed when excluding those with referral indications associated with CSC involvement. Consistent guidance and a strengths-based family approach is needed to address referral disparities for this group of women and birthing people.

**Supplementary Information:**

The online version contains supplementary material available at 10.1186/s12884-025-08197-5.

## Background

Pregnancy is often considered a ‘window of opportunity’ to identify medical and psycho-social needs and signpost families to appropriate services [1–3]. Through frequent antenatal contact with pregnant women and birthing people, midwives are in a unique position to obtain a wider picture of the social circumstances in which a child will come into the world. Under UK children’s safeguarding legislation, midwives and other professionals have a duty to send a referral to local child services when they have concerns regarding parental abilities to care safely and adequately for the newborn child. Such a referral can trigger the involvement from Children’s Social Care (CSC, the term used for child protection agencies in the UK) through a process of ‘pre-birth assessment’, and subsequent child protection interventions might be put in place to safeguard the child. Safeguarding interventions range from voluntary support and statutory child protection plans, to court-ordered separation after birth in the most concerning cases [4]. In England, pre-birth referral and assessment guidance is issued by local community authorities. A previous study highlighted different thresholds and standards of practice across 147 Local Safeguarding Children Boards in England, inferring the potential for individual discretion and a risk of bias in professional referrals [5]. 

On a par with global trends in child protection rates, UK data over the last decade have shown increased rates of CSC involvement among children across all age groups, including pre-birth assessments and infants with CSC involvement [6]. The UK Office of National Statistics reported that in 2023 among approximately 565,000 births in England almost 8,000 unborn babies and 21,000 infants under the age of one were considered by CSC to be at risk of harm and required some level of intervention [6, 7]. Poverty-driven vulnerabilities are recognised as an important reason behind these increasing numbers [8–12] yet cannot explain the racial and ethnic disparities among children known to CSC [10], with children from Black and mixed ethnic backgrounds being overrepresented in referrals to child welfare services in many high income countries [13–16]. A study from a UK inner-city population (Liverpool) found that children with pre-birth CSC contact were more likely to end up in state care during childhood. They also found that Black and Asian children, while referred at older ages than White children, were more likely to enter the care system [17]. These ethnic disparities are mirrored in other maternal and child health indicators, such as the higher mortality and morbidity among UK Black and Asian women [18, 19]. 

The use of administrative data for research purposes has become increasingly popular and accessible. Previous work by Ireland et al. (2024) into the social and health characteristics of mothers who face court-ordered removal of their child(ren) has used linked databases from Family Courts (CAFCASS), hospital admission data for England and a mental health services database in South London [20]. It has shone a light on those women with the highest level of CSC involvement, i.e. court-ordered removal. Yet, information about pregnancy care of these mothers and characteristics of the wider group of women in contact with CSC, including those with voluntary offers of support and child protection plans, remains limited. To address this evidence gap, and by extension, address disparities in CSC involvement, it is important to interrogate referrals to and contact with CSC at the earliest stages, i.e. in utero, in the context of maternity care.

### Study aims

The aims of this study therefore were:

1. to describe the demographic, socio-economic and clinical characteristics of women who have contact with CSC during pregnancy compared to those without;
2. to examine whether those in contact with CSC during pregnancy presented with referral indications concordant with local pre-birth assessment guidance for referral;

For the latter, we adopted the hypothesis that those with referral indications identified in the database will have contact with CSC. By extension, we hypothesised that those without any identifiable referral indications would not be in contact with CSC.

This information is fundamental for health and social care professionals to deliver equitable care and support, and address bias in CSC contact at this critically vulnerable window of development.

### Patient and public involvement

This study is a component of a doctoral research study (MUMS@RISC, NIHR302565). The MUMS@RISC advisory group, with six women with lived experience of CSC involvement in pregnancy, was consulted at regular intervals, helping to identify areas of concern. Discussions on study design, and preliminary findings, through their lived experience, contributed towards interpretation of findings.

## Methods

### Study design, setting and cohort formation

This was a retrospective cross-sectional study using data from the Early Life Cross Linkage in Research (eLIXIR-Born in South London, eLIXIR-BiSL) cohort. The eLIXIR-BiSL dataset links pseudonymised electronic health records from two large maternity care providers and one mental health care provider within the National Health Service (NHS) in South London [21]. This urban area has some of the highest levels of ethnic and socio-economic diversity in the UK. Data are provided by opt-out consent, with necessary ethical and information governance approvals, and the linkage is hosted by the Clinical Records Interactive Search (CRIS) Trusted Research Environment.

Maternity data are recorded on the BadgerNet (CleverMed) maternity platform during antenatal contacts, intrapartum care and the early postpartum period. Mental health data from treatment episodes at the South London and Maudsley NHS Foundation Trust are obtained through CRIS [22]. In the UK, there is a distinction between primary mental health services (with access through self-referral and offering low intensity psychological interventions, such as Cognitive Behavioural Therapy) and secondary mental health services, for those with moderate to severe or complex mental health needs, requiring psychiatric input from multi-disciplinary teams after a referral from a general practitioner or other first-line healthcare professionals. Therapy can be based in the community or in inpatient psychiatric units. If a person has ever received treatment from the designated secondary mental health service in South London, they will have a CRIS record, in which multiple care episodes will be recorded . It is important to note that only those requiring treatment in secondary mental health services will have a CRIS record, and absence of a CRIS record therefore does not equate to missingness. Maternity and mental health data is linked at regular intervals per pregnancy episode, using unique identifiers, the providing a repository of real-time, anonymised, structured data with the addition of approximately 16,000 pregnancies per year.

**Fig. 1:**
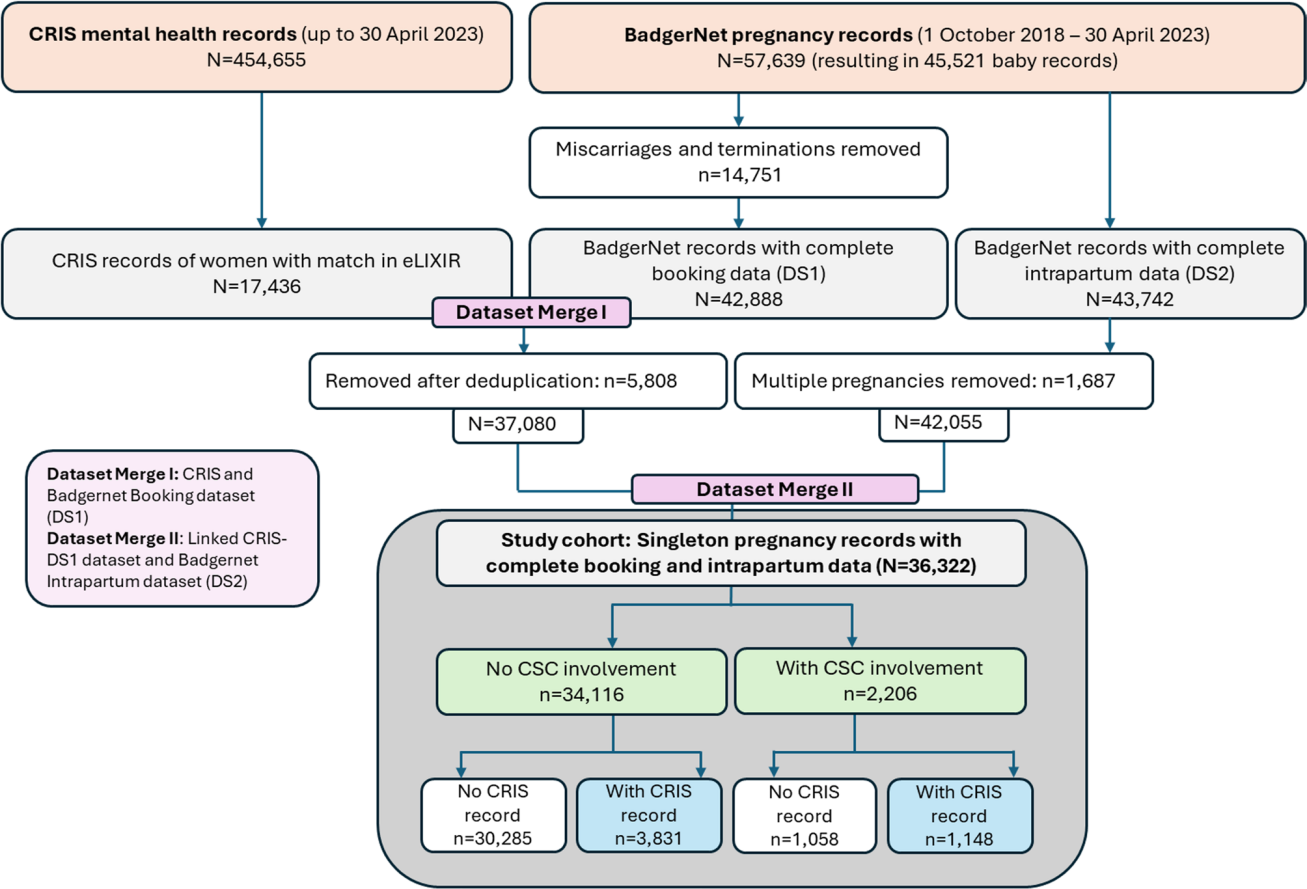
Cohort composition

For this study, a cohort of 57,639 BadgerNet pregnancy records (resulting in 45,521 babies) was used, from whom data were collected between 1 October 2018 and 30 April 2023. Figure 1 displays the cohort composition in more detail. We identified all pregnancy records with complete booking data, with their linked CRIS mental health record where relevant. Linked records which had complete intrapartum data and resulting in a singleton birth, including livebirth, late pregnancy loss (born with no signs of life at or after 24 weeks of pregnancy), or early neonatal death (death during the first 28 days of life) were retained. Pregnancy records resulting in multiple births were excluded to avoid introducing bias in the cohort in view of compounded vulnerability when two or more babies enter a family at the same time. Pregnancy records resulting in miscarriage or termination were also excluded, as pre-birth assessments tend to be initiated at later stages of pregnancy.

### Outcome of interest: children’s social care (CSC) contact in pregnancy

The outcome of interest was current contact with CSC during the pregnancy episode. This information was retrieved through variables from the maternity record, as to date there is no linkage available between maternity records and CSC records in the designated local area. For the purpose of this study, CSC contact was defined as initial or ongoing contact during pregnancy for the unborn foetus, and may have ranged from a referral to CSC, voluntary offers of support (S. 17, for instance financial or practical support) to mandatory child protection interventions (S. 47), in line with correspondent sections of the Children’s Act 1989 [4]. 

**Fig. 2:**
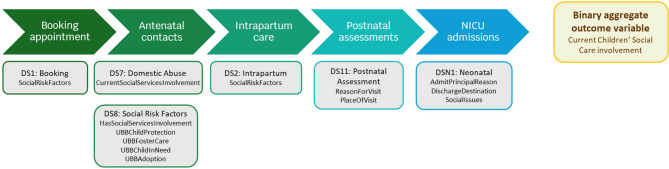
Exposure of interest: contact with Children’s Social Care (CSC)

CSC contact in pregnancy is a dynamic process and can be documented at different antenatal contacts. We searched different datasets within the maternity database to identify the various timepoints when midwives assess social risk factors, and the need for CSC referral and involvement (Fig. 2). We subsequently created an aggregate binary variable consisting of information on CSC contact across the pregnancy record at any point. This included any pregnancy records during which a referral to CSC was made, even when maternity staff did not have further information about the outcome of the pre-birth assessment undertaken by local child protection agencies. We did not account for intensity of CSC involvement during pregnancy due to the variable nature of child protection processes, whereby involvement can fluctuate over time.

### Covariates of interest

For our first study aim, we retrieved information about maternal baseline demographic and clinical characteristics. These are recorded during the first antenatal contact (the ‘booking’ appointment) when midwives request information on socio-economic background, and past and current medical and obstetric history. Socio-demographic covariates included maternal age, country of birth, ethnicity, socio-economic status, relationship status, employment status, accommodation status, and the need for an interpreter and were retrieved from the maternity record. Ethnicity data were grouped as five aggregated categories used in the UK Census classification (Asian, Black, Mixed or multiple ethnicity, Other or White) [23]. Socio-economic status was derived from quintiles of the Index of Multiple Deprivation (IMD) [24]. 

Baseline clinical characteristics included parity, gestational age at booking, maternal BMI, smoking status, previous abortions, medical (such as pre-existing diagnosis of chronic conditions, clotting disorder, etc.) and obstetric risk factors (such as previous postpartum haemorrhage, foetal growth restriction, gestational diabetes, etc.), presence of mental health problems, previous drug use, and disclosure of previous domestic abuse. Late initiation of antenatal care (‘late booking’) was identified in accordance with the NHS key performance indicator for antenatal care to be initiated before 13 weeks’ gestation.

### Previous CSC involvement

Mothers with historic CSC contact are more likely to have repeat episodes of CSC involvement [25]. Therefore, we included previous CSC involvement through an aggregate variable capturing information from antenatal enquiry about previous contact with CSC . In addition, where women had multiple pregnancy episodes recorded on the maternity record, an index episode of CSC involvement was transferred to subsequent pregnancies as ‘previous CSC involvement’.

### Reasons to refer in accordance with local guidance

For our second study aim, we identified indications to refer to CSC from the pre-birth assessment guidance provided in the London Safeguarding Children Partnership’s pre-birth referral and assessment guidance [26]. Some of these referral indications were impossible to retrieve from the pregnancy record, for instance if a parent or other adult in the household had been identified as posing a risk to children (for instance because they were on the Sex Offenders’ Register), or if a parent was previously suspected of fabricating an illness in a child. For 12 of the 14 referral indications, we were able to extract corresponding variables from both maternity and mental health data sources or used a combination of variables across different datasets to match each referral indication. Referral indications were also sense-checked with a group of women with lived experience and with maternity care professionals, to ensure these were representative of clinical practice. We adopted a conservative approach, to avoid over-identifying risk factors in the dataset. For example, where the guidance stipulates to refer in case of ‘mental health problems that might impact on the care of a child’ [26], we only included those pregnancy records where the mother was detained under the Mental Health Act or treated by specialist mental health teams (e.g. addiction, forensic or child and adolescent mental health services). A full overview of reasons to refer, and corresponding variables can be found in Supplementary Table S2.

### Statistical analyses

For the first objective, incidence of each demographic, socio-economic and clinical variable was calculated as number and percentage for women with and without CSC contact. For each binary or categorical variable, a χ^2^ test was used to compare frequencies between the two groups. For continuous variables, characteristics were compared using t-tests, and reported as mean (SD), with the cohort comparison as mean difference (95% Confidence Interval, CI). Age was evaluated as a continuous and categorical variable, with stratified age groups.

For the second objective, we investigated the associations between each of the referral indications and the outcome of interest (CSC contact) by estimating unadjusted Risk Ratios (RR) for each of the referral indications, through binomial regression models using the Stata command ‘binreg, rr’. We used a standard method [27, 28] to correct the standard errors for possible clustering of multiple pregnancy episodes by mother, using the Stata command ‘cluster’.

As part of our second objective, we conducted further analysis to examine the cohort of women without any identifiable CSC referral indications in more depth. Similar to our first research objective, we used descriptive statistics to compare baseline characteristics between those with and without CSC contact, applying χ^2^ tests for categorical variables. Unadjusted Risk Ratios for key demographic variables, in the absence of referral indications were calculated using the Stata command ‘binreg, rr’. The same method of adjusting for multiple pregnancy episodes by mother was applied as described above.

We assessed the extent of missing data and reported missingness on key demographic variables in Tables 1 and 3. For variables pertaining social risk factors, for instance current substance use, we considered missingness as an absence or non-identification of risk. The level of missingness was low for most variables (< 5%), except for relationship status.

Stata (version 18.0, StataCorp, College Station, TX, USA) was used for data manipulation and statistical analysis. Significance was set at a p-value of < 0.05.

## Results

After deduplication and merging of datasets, 36,322 pregnancy records with complete booking and intrapartum data of singleton pregnancies were retained (Fig. 1). There were 2,206 (6%) records documenting CSC involvement.

### Study aim 1 - sample characteristics

The demographic, socio-economic and clinical characteristics of the sample are described in Table 1. Pregnancy records with CSC contact had a recorded maternal age at booking that was on average 3.5 years younger than those without and were from women with lower socio-economic status. This was reflected in overrepresentation from the two lowest IMD quintiles, and by higher rates of social housing and unemployment. Pregnancies with CSC contact were more frequently of UK-born and single women. Pregnancies of Black women and those of mixed or multiple ethnic groups were overrepresented, in contrast to those of White or Asian women.

When comparing clinical characteristics, CSC contact was less frequently observed in first pregnancies (nulliparous women), with higher proportions of smoking and medical and obstetric risk factors. Mental health problems were more frequently observed in pregnancies with CSC contact, as well as previous drug use and previous domestic abuse. Pregnancy records with CSC contact were of women who had more frequently undergone three or more abortions prior to the pregnancy episode, and initial contact with maternity services tended to occur later in the pregnancy.

**Table 1:** Maternal baseline characteristics of pregnant women with and without children’s social care involvement

	No CSC contact during pregnancy (*n* = 34,116)	CSC contact during pregnancy (*n* = 2,206)
**Demographic and socio-economic characteristics**		
Maternal age at booking (years) mean ± SD	32.9 (5.18 SD; 32.9–33.00 95%CI)	29.3 (7.03SD; 29.0–29.5 95%CI)
Maternal age, by age category		< 0.001^*^
16y or younger	3 (0.01%)	4 (0.18%)
17-19y	250 (0.73%)	196 (8.88%)
20-24y	2,048 (6.00%)	445 (20.17%)
25-34y	18,332 (53.73%)	998 (45.24%)
35-39y	10,355 (30.35%)	394 (17.86%)
40y or older	3,128 (9.17%)	169 (7.66%)
Parity at booking		< 0.001
P0	18,483 (54.18%)	737 (33.41%)
P1	10,168 (29.80%)	588 (26.65%)
P2	33,558 (10.43%)	424 (19.22%)
P3	1,203 (3.53%)	261 (11.83%)
P4	427 (1.25%)	109 (4.94%)
P5 or more	277 (0.81%)	87 (3.94%)
Born in the UK, *n* (%)	14,342 (42.04%)	1,257 (56.98%)
Ethnicity, as per ONS categories *n* (%)		< 0.001
White	17,742 (52.00%)	741 (33.59%)
Asian/Asian British/Asian Welsh	3,455 (10.13%)	122 (5.53%)
Black/Black British/Black Welsh/African/Caribbean	6,954 (20.38%)	918 (41.61%)
Mixed or multiple ethnic groups	1,672 (4.90%)	219 (9.93%)
Other Ethnic Group	2,461 (7.21%)	117 (5.30%)
Missing	1,832 (5.37%)	89 (4.03%)
Social deprivation (Index of Multiple Deprivation, IMD) Quintile		< 0.001
1 (most deprived)	6,432 (18.85%)	641 (29.06%)
2	13,847 (40.59%)	1,036 (46.96%)
3	8,559 (25.09%)	369 (16.73%)
4	3,280 (9.61%)	98 (4.44%)
5 (least deprived)	1,391 (4.08%)	26 (1.18%)
missing	607 (1.78%)	36 (1.63%)
Relationship status		< 0.001
Married/partner/cohabiting	17,356 (50.87%)	397 (18.00%)
Single	10,438 (30.60%)	1,327 (60.15%)
Separated/divorced/widowed	186 (0.55%)	24 (1.09%)
Missing	6,136 (17.99%)	458 (20.76%)
Employment status		< 0.001
Employed^**^	25,669 (75.24%)	887 (40.21%)
Unemployed	4,343 (12.73%)	896 (40.62%)
Housewife/carer	2,125 (6.23%)	182 (8.25%)
In education	638 (1.87%)	127 (5.76%)
No rights to work	159 (0.47%)	17 (0.77%)
Other^+^	183 (0.55%)	29 (1.31%)
Missing	999 (2.93%)	68 (3.08%)
Accommodation		< 0.001
Own property	13,455 (39.44%)	154 (6.98%)
Private rental	9,887 (28.98%)	289 (13.10%)
Social housing (through council or housing association)	5,612 (16.45%)	1,124 (50.95%)
Temporary accommodation, incl. family or friends	2,828 (8.29%)	448 (20.31%)
No fixed abode	86 (0.25%)	29 (1.31%)
Other^++^	390 (1.14%)	73 (3.31%)
Missing	1,858 (5.45%)	89 (4.03%)
Interpreter Needed	2,526 (7.40%)	120 (5.44%)
**Clinical and medical characteristics**		< 0.001
Gestational age at booking (days), (mean, SD, 95%CI)	87.5 (49.9 SD; 95%CI 87.0-88.1)	100.2 (57.1 SD; 95%CI 97.8-102.6)
Late booking > 13wks^#^	5,234 (15.34%)	578 (26.20%)
Body Mass Index (kg/m^2^) at booking, (mean, SD, 95%CI)	25.5 (5.4 SD, 95% CI 25.4–25.6)	27.9 (7.3 SD, 95% CI 27.6–28.2)
3 or more previous abortions prior to booking	1,669 (4.89%)	285 (12.92%)
Smoker at booking	906 (2.66%)	413 (18.72%)
Previous drug use disclosed	1,899 (5.57%)	385 (17.45%)
Previous domestic abuse disclosed	1,610 (4.72%)	1,137 (51.54%)
Medical risk factors identified during pregnancy	23,440 (68.71%%)	1,863 (84.45%)
Obstetric risk factor identified during pregnancy	11,488 (33.67%)	1,033 (46.83%)
Mental health problems reported during pregnancy	9,313 (27.30%)	1,260 (57.12%)
Known to Secondary Mental Health Services	3,831 (11.23%)	1,148 (52.04%)

* *P*-values reflect significance of differences between no CSC involvement group versus CSC group

**Includes self-employment, being on maternity leave and being furloughed during COVID-19 pandemic as both imply contractual employment

+ Includes voluntary work, medically unfit, intentional career break, retired

++ Includes UK Border Agency accommodation, psychiatric unit stay, accommodation through church, university halls, caravan site, supported accommodation, army accommodation

^#^ Excludes any late bookings due to transfer of care from different hospital

### Study aim 2 – indications for CSC referral versus actual CSC contact

**Fig. 3:**
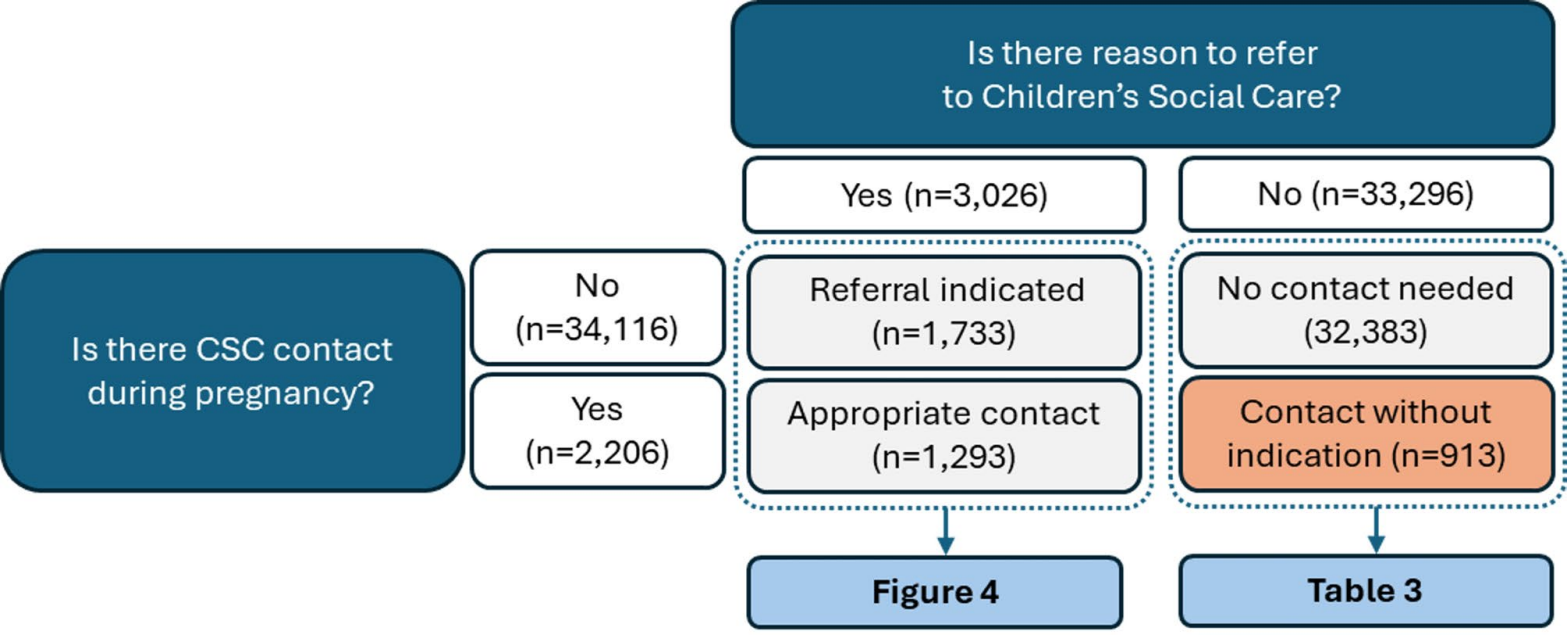
Referral indications versus actual CSC contact

For the second objective, we hypothesised that all those in contact with CSC would have at least one or more indication for referral in line with local guidance. Conversely, we hypothesised that all those without indications for referral, would not be in contact with CSC. Figure 3 shows both our hypotheses were not upheld: (1) We identified a total of 3,026 pregnancies with one or more indications for CSC involvement, whereas only 1,293 of those had contact with CSC recorded; (2) We identified 913 pregnancy records without an indication for CSC contact but with recorded CSC contact.

### Referral indications identified

**Fig. 4:**
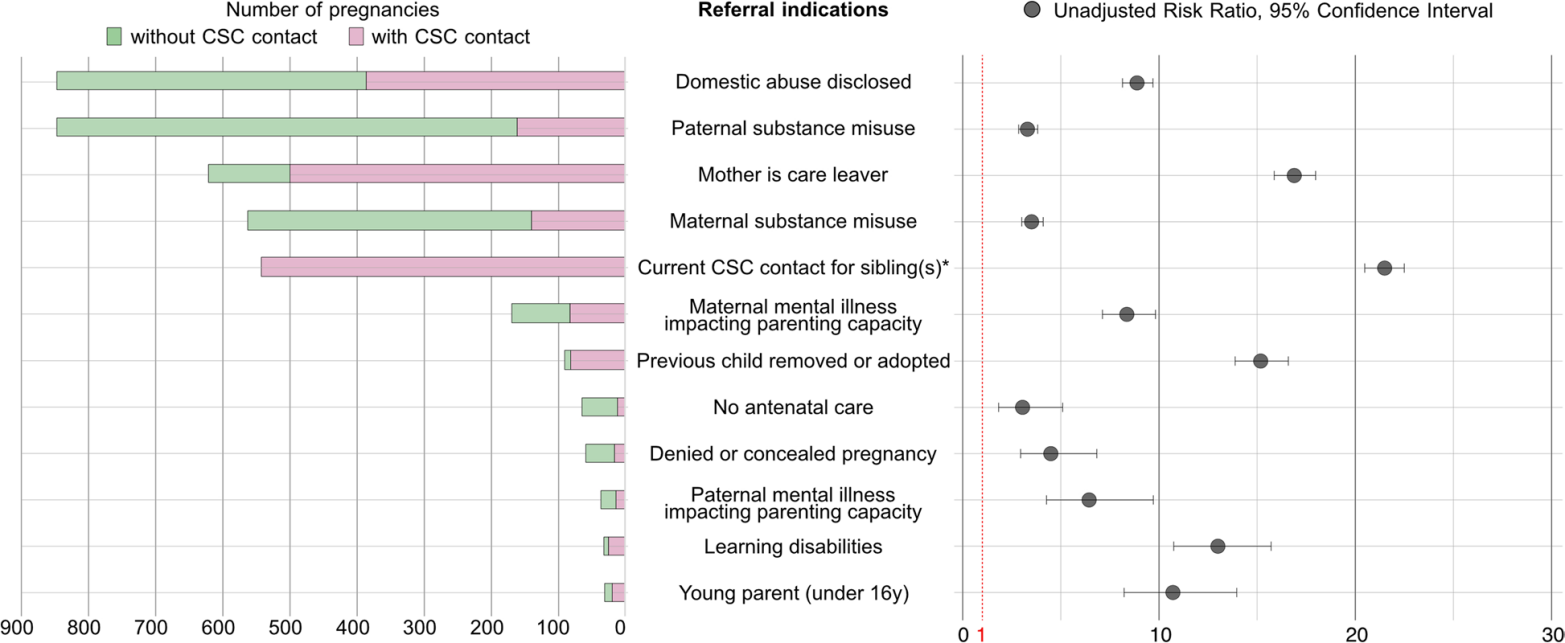
Referral indications and actual contact, in frequencies and unadjusted risk ratios

For those with an indication for referral, we explored to what degree each of the indications for referral was associated with actual CSC contact. A visual overview of referral indications, with frequencies, proportions of actual CSC contact and unadjusted RRs is shown in Fig. 4. Full details are available in Table 2.

**Table 2:** Referral indications versus actual CSC contact

	No CSC contact *n* = 34,116 Frequency (%)	With CSC contact *n* = 2,206 Frequency (%)	Total pregnancy records with referral indication	Percentage of pregnancy records with identified referral indication, with CSC involvement	Unadjusted Risk Ratio (95% CI)	*p*-value
Previous child removed or adopted	10 (0.03%)	81 (3.67%)	91	89.01%	15.18 (13.88–16.59)	< 0.001
Current CSC contact for older sibling(s)^*^	0	542 (100%)	542	100%	21.50 (20.52–22.53)	< 0.001
Maternal severe mental illness impacting parenting capacity^○^	86 (0.25%)	83 (3.76%)	169	49.11%	8.36 (7.12–9.82)	< 0.001
Paternal severe mental illness impacting parenting capacity^+^	22 (0.06%)	14 (0.63%)	36	38.89%	6.43 (4.27–9.71)	< 0.001
Maternal substance use	423 (1.24%)	140 (6.35%)	563	24.87%	4.30 (3.69–5.02)	< 0.001
Paternal substance use	686 (2.01%)	161 (7.30%)	841	19.01%	3.29 (2.85–3.82)	< 0.001
Young parent (under 16y)	11 (0.03%)	20 (0.91%)	31	64.52%	10.71 (8.22–13.96)	< 0.001
Domestic abuse disclosure either in SLaM or Maternity	461 (1.35%)	386 (17.50%)	847	45.57%	8.88 (8.14–9.69)	< 0.001
Learning disabilities impacting parenting capacity	7 (0.02%)	25 (1.13%)	32	78.12%	13.00 (10.75–15.71)	< 0.001
Mother is a care leaver	120 (0.35%)	500 (22.71%)	621	80.68%	16.89 (15.87–17.98)	< 0.001
No antenatal care	53 (0.16%)	12 (0.54%)	65	18.46%	3.05 (1.83–5.09)	< 0.001
Denied or concealed pregnancy	43 (0.13%)	16 (0.73%)	59	27.12%	4.49 (2.95–6.83)	< 0.001

^*^ Unadjusted risk ratio could not be estimated using Stata binreg command, in view of 100% of cases with CSC contact. For this particular referral indication, we used the Stata cs command instead. Stata binreg and cs commands gave comparable unadjusted risk ratios for other referral indications. However, the cs command does not allow for adjusting for clustering of pregnancies by mother for this referral indication

^○^ Maternal severe mental illness was defined as either being detained under the Mental Health Act or treated by specialist mental health teams (e.g. addiction, forensic or child and adolescent mental health services), and excluded treatment by perinatal mental health community team

^+^ Paternal severe mental illness was defined as schizophrenia and psychosis

Referral indications related to any form of previous contact with CSC were strongly associated with CSC contact in the current pregnancy: women who had a previous child adopted or removed were 15 times more likely to have current CSC contact than those without this history. Women who had been in state care themselves as a child, another indication, were 17 times more likely of having CSC contact during their pregnancy. All of those with ongoing CSC involvement for older children had CSC involvement during this pregnancy. Disclosure of current domestic abuse (DA) and paternal substance use were the most frequently identified indications to refer. For pregnancies with DA disclosure, mothers were almost 9 times more likely to have contact with CSC compared to pregnancies without. However, only 45.6% of those with a DA disclosure were in contact with CSC. Similar discrepancies between detection of referral indications and actual contact with CSC was observed for paternal and maternal substance use, not seeking antenatal care and concealing a pregnancy. Overall, those with more risk factors recorded were more likely to have CSC contact, with all 11 pregnancies that had 5 risk factors having been in contact with CSC. Conversely, only 35.0% of pregnancies with a single referral indication had CSC involvement (uRR 12.76, 95%CI: 11.72–13.89, p-value < 0.001) (Supplementary Table S3).

### No referral indications identified

As shown in Fig. 3, referral indications could not be identified in 913 of the 2,206 pregnancy records with CSC contact, prompting further investigation in a post-hoc analysis. We wanted to examine if and how those with CSC involvement (in the absence of identifiable referral indications) differed from those that did not have CSC involvement (again, in the absence of referral indications). A total of 33,296 pregnancy records were retained for this analysis, all without any identifiable referral indications for CSC contact, of which 913 had CSC involvement (2.74%) (See Table 3). The comparison group consisted of all pregnancy records where no referral indications had been identified and without any CSC contact (*n* = 32,383).

**Table 3:** Covariates associated with CSC involvement when there is no identifiable referral indication (*n* = 33,296)

	No CSC involvement (*n* = 32,383)	CSC involvement during pregnancy (*n* = 913)	Unadjusted RR (95%CI)	*p*-value
Maternal age at booking+				< 0.001
17-19y	191 (0.59%)	59 (6.46%)	10.05 (7.86–12.85)	< 0.001
20-24y	1,860 (5.74%)	160 (17.52%)	3.37 (2.82–4.03)	< 0.001
25-34y	17,387 (53.69%)	418 (45.78%)	Reference	-
35-39y	9,933 (30.67%)	198 (21.69%)	0.83 (0.70–0.99)	0.036
40y or older	3,012 (9.30%)	78 (8.54%)	1.08 (0.84–1.37)	0.557
Born in the UK, n (%)	13,426 (41.46%)	468 (51.26%)	1.52 (1.33–1.74)	< 0.001
Ethnicity, as per ONS categories n (%)				< 0.001
White	16,866 (52.08%)	295 (32.31%)	Reference	-
Asian/Asian British/Asian Welsh	3,365 (10.39%)	60 (6.57%)	1.02 (0.76–1.36)	0.897
Black/Black British/Black Welsh/African/Caribbean	6,546 (20.21%)	385 (42.17%)	3.23 (2.77–3.76)	< 0·001
Mixed or multiple ethnic groups	1,526 (4.71%)	83 (9.09%)	3.00 (2.35–3.82)	< 0·001
Other Ethnic Group	2,340 (7.23%)	54 (5.91%)	1.31 (0.97–1.77)	0.076
Missing	1,740 (5.37%)	36 (3.94%)	-	-
Social deprivation (IMD) Quintile				< 0.001
1 (most deprived)	6,084 (18.79%)	251 (27.49%)	7.65 (3.61–16.18)	< 0·001
2	13,088 (40.42%)	439 (48.08%)	6.26 (2.97–13.19)	< 0·001
3	8,150 (25.17%)	149 (16.32%)	3.47 (1.63–7.35)	0·001
4	3,145 (9.71%)	49 (5.37%)	2.96 (1.34–6.53)	0·007
5 (least deprived)	1,344 (4.15%)	7 (0.77%)	Reference	-
missing	572 (1.77%)	18 (1.97%)	-	-
Relationship status				< 0.001
Married/partner/cohabiting	16,781 (51.82%)	216 (23.66%)	Reference	-
Single	9,653 (29.81%)	498 (54.55%)	3.86 (3.28–4.54))	< 0·001
Separated/divorced/widowed	172 (0.53%)	14 (1.53%)	5.92 (3.51–9.99)	< 0·001
Missing	5,777 (17.84%)	185 (20.26%)	-	-
Employment status				< 0.001
Employed^*^	24,448 (75.50%)	411 (45.02%)	Reference	-
Unemployed	4,016 (12.40%)	329 (36.04%)	4.58 (3.97–5.28)	< 0.001
Housewife/carer	2,062 (6.37%)	75 (8.21%)	2.12 (1.66–2.71)	< 0.001
In education	586 (1.81%)	40 (4.38%)	43.68 (2.80–5.33)	< 0.001
No rights to work	142 (0.44%)	7 (0.77%)	2.84 (1.37–5.89)	< 0.005
Other^++^	171 (0.53%)	11 (1.20%)	3.66 (2.05–653)	< 0.001
Missing	958 (2.96%)	40 (4.38%)	-	-
Parity at booking				< 0.001
P0	17,359 (53.61%)	283 (31%)	Reference	-
P1	3,792 (30.24%)	253 (27.71%)	1.57 (1.33–1.85)	< 0.001
P2	3,408 (10.52%)	195 (21.36%)	3.37 (2.82–4.03)	< 0.001
P3	1,147 (3·54%)	112 (12.27%)	5.55 (4.49–6.85)	< 0.001
P4	411 (1.27%)	38 (4.16%)	5.28 (3.81–7.31)	< 0.001
P5 or more	266 (0.82%)	32 (3.50%)	6.69 (4.70–9.53)	< 0.001
Previous domestic abuse disclosed	1,246 (3.85%)	373 (40.85%)	12.72 (11.21–14.43)	< 0.001
Previous social services involvement**	172 (0.53%)	382 (41.84%)	42.52 (38.42-47-06)	< 0.001
Lifetime contact with Secondary Mental Health Services	3,191 (9.85%)	352 (38.55%)	5.27 (4.62–6.01)	< 0.001

+ Excludes under 16y olds as this was an indication for CSC involvement during pregnancy

++ Includes voluntary work, medically unfit, intentional career break, retired

* Includes self-employment, being on maternity leave and being furloughed during COVID-19 pandemic as both imply contractual employment

**This excludes any ongoing CSC involvement for siblings or previous involvement whereby children were removed from parental care (), or previous involvement for the mother herself as a child (mother is a care leaver), as these were both indications for CSC involvement in the current pregnancy

Pregnancies with no identifiable referral indication for CSC contact were more common among unemployed women, and those that were single, separated, widowed or divorced. Younger women (under 25 year) were more often in contact with CSC, especially those aged 17 to 19. Black women were three times as likely to have contact with CSC than White women, and contact was also higher among women from mixed or multiple ethnic groups. Compared to nulliparous women, multiparous women were more likely to have CSC contact, with congruency with increased parity. Those with a history of CSC contact had the highest risk for contact during the current pregnancy episode, in the absence of current risk factors. In our model, we excluded those instances where a child had been removed from parental care in the past or where the mother had been in care herself, as these were indications for current involvement according guidance. Therefore, previous CSC in this analysis only included historic episodes of CSC involvement where the child had remained in parental care and involvement had ceased prior to booking. Of the other covariates, both lifetime contact with secondary mental health services and previous DA disclosures were also significantly associated with CSC contact.

## Discussion

### Main findings

This study identified significant differences between those in contact with CSC and those without, for a wide range of demographic, clinical and medical characteristics. Those in contact with CSC during pregnancy were more frequently younger, multiparous women, from Black or mixed ethnic backgrounds and living in areas with the highest levels of deprivation. Women in contact with CSC had more often medical, obstetric and psychiatric problems during pregnancy. They more frequently smoked in pregnancy and initiated antenatal care later.

When examining the reasons for CSC contact, we found that not all indications for CSC contact triggered a referral to the same extent. Referral indications pertaining previous CSC contact seemed to have the strongest association with current CSC contact. Referral indications related to paternal issues (either paternal substance use or paternal mental illness) or minimal antenatal care (either no antenatal care or concealing a pregnancy) had the weakest association with CSC contact.

Our further investigations into those without any identifiable referral indications showed a strong association between CSC contact and socio-economic and ethnic covariates as well as prior contact with mental health and social services.

When reviewing our findings in the context of the wider literature around CSC involvement, we found that in this urban area with high levels of socio-economic and ethnic diversity, women with CSC involvement were more often from younger age groups, yet were also more likely to be multiparous. This echoes previous evidence demonstrating young women aged 16 to 19 years are overrepresented in those with court-ordered infant removal [20] and most at risk of having recurrent CSC involvement in subsequent pregnancies [25]. While uRRs for older age groups in our study were no longer significant, Broadhurst et al. (2015) found increased age reduced the likelihood of recurrent care proceedings, suggesting that maturation plays a role in reducing the likelihood of CSC involvement [25]. Societal biases and judgement towards who is ‘fit’ to mother might further influence higher rates of young mothers in contact with CSC.

Age and parity were not the only characteristics where we identified disparities in CSC contact. We found that anything other than conventional employment was associated with CSC contact, with or without referral indications identified. Deprivation, whether this was approached through employment status or IMD quintiles, was consistently found to be strongly associated with CSC involvement. The association between socio-economic status and CSC involvement has been widely evidenced in literature on child welfare interventions [10–12, 16, 20]. However, CSC contact has to be seen through an intersectional lens, whereby disadvantage is not the result of a single axis (for instance poverty) but the product of intersecting vulnerabilities or identities (e.g. race, being a single parent) [29]. The intersectionality between ethnicity and poverty in the context of CSC involvement has been widely reported [30–32]. UK studies have consistently shown ethnic disproportionality in CSC interventions, with an overrepresentation of Black African and Black Caribbean children in care, and an under-representation of South Asian children, relative to their proportions in the wider population [11, 33]. However, after adjusting for poverty and deprivation, ethnic disproportionality changes significantly in many of these studies, as certain ethnic groups face higher levels of deprivation, in particular Black African and Black Caribbean populations [10, 16, 34]. Our findings reflect such intersectionality, with disproportionate numbers of women from Black or mixed ethnic backgrounds having CSC contact, as well as women from the poorest areas in South London. Significance of these disparities remained among women with no identifiable referral indications and resembles a social gradient in child welfare interventions that has been previously described [10]. Our findings need to be viewed in the wider literature of ethnic disproportionality in CSC involvement throughout childhood and adolescence: with ethnic disparities in CSC involvement already existing prior to birth, these are likely to be amplified during childhood, as children from Black and mixed ethnic backgrounds tend to be referred at later stages [17] and therefore disproportional representation might be further amplified over time.

In addition to socio-demographic disparities, our data highlighted a cycle of CSC involvement, whether this was during women’s own childhood or for their older children. The pre-birth assessment guidance we consulted stipulated that (1) being a ‘care-leaver’, (2) having previous children adopted or fostered, (3) having older children with ongoing CSC involvement were among the indications for referral to CSC. Other indications inconsistently prompted CSC contact, yet these three indications had the strongest association with CSC involvement in pregnancy. Growing up in the care system has been associated with lifelong poorer health, education and employment outcomes [35], but this does not justify a one-size-fits-all approach to automatic referrals for care leavers. Such an approach does not consider individual circumstances, strengths and support systems, and considers ‘care experience’ in itself as a safeguarding concern. To address discrimination and stigmatisation of people with care experience, the Independent Review of Children’s Social Care recommended to make care experience an additional ‘protected characteristic’ in line with existing UK equality legislation [36]. Across England, local authorities have followed suit, but if pre-birth assessment guidance continues to consider care experience as a safeguarding risk, discrimination will persist, and the intergenerational cycle of CSC involvement will remain.

We found congruency between the number of identified referral indications and increased likelihood of CSC contact, suggesting that those with accumulative risk factors are adequately being referred. However, we found that some referral indications have lower RRs than others, raising questions about maternity professionals’ decision-making as to which single risk factors require escalation to CSC. Particularly concerning is the inconsistent escalation after domestic abuse disclosures, with only half of disclosures prompting a referral to CSC. Professionals’ apprehensiveness towards DA enquiry, risk recognition and escalation to appropriate support services in this context has been well documented and might provide an explanation for these findings. This has been evidenced by recent work, evidencing that routine enquiry about DA was missing from medical records among pregnant women with cumulative risk factors, and limited support was made available [37]. 

### Strengths and limitations

This study is the first to investigate contact with CSC during pregnancy through the use of linked pregnancy and mental health records, and to compare CSC contact with local pre-birth assessment guidance. As our study was undertaken in the ethnically and socio-economic diverse urban area of South London, our findings might not be generalisable to more rural areas, or regions with less diversity in the UK and other high-income countries. By using linked NHS healthcare records, we minimised the risk of selection and surveillance bias, as data were collected independently of the research hypothesis by midwives and obstetricians in maternity settings. While this is a strength, the availability and quality of data was dependent on clinicians’ accuracy in reporting information. One such area of concern pertains the recording of ethnicity within administrative and healthcare record data [38]. Our analysis of ethnicity as covariate was limited by the available categories within the dataset. It is also unclear to what extent recorded ethnicity aligns with self-identification, and whether errors in ethnicity data are present due to healthcare professionals’ assumptions or women’s reluctance to report ethnicity, due to fear of data sharing and/or discrimination. Another area of concern when using healthcare record data is around professional confidence to ask about sensitive issues, such as domestic abuse, mental health, previous social care involvement. Domestic abuse enquiry in particular has previously been highlighted as an issue in maternity care settings and is known to be underreported, including in this database [39]. Furthermore, there might be relevant information pertaining the woman’s ongoing psychosocial situation that is not captured in maternity records. Possible incomplete or inaccurate identification of risk factors is therefore a limitation of the study, especially as not all referral indications could be retrieved through the variables at hand. This means that our study might have overestimated the number of ‘unfounded’ referrals, i.e. pregnancy records with CSC contact but without any identifiable referral indication. Another limitation is that it was not possible from the dataset variables to differentiate between Children’s or Adult Social Care, i.e. support services focusing on vulnerable adults. Current practice would stipulate that if women were referred to Adult Social Care themselves, inevitably their unborn child would also be referred to Children’s Social Care. Distinction between the two is impossible to make in the dataset, so we assumed that social care variables referred to “Children’s Social Care”.

### Implications for practice, research and policy

This study has highlighted the need for careful professional consideration about who needs to be in contact with CSC. At all times, safeguarding risk assessments need to be grounded in the present, taking into account a holistic, strengths-based and family approach, with appropriate consideration for adverse life events and their enduring impact. Especially for those living in poverty and from ethnic diverse communities, decisions around CSC referrals have to be made without bias or judgement, but based on current safeguarding risks towards the unborn baby. CSC have an important role to play to support families to provide a safe and stable home for their children. This also means that those with referral indications that are currently not in touch with CSC, have to be appropriately referred when required to do so. Further research needs to explore the social gradient in CSC contact during pregnancy, by examining causality between certain socio-economic and demographic maternal characteristics and CSC contact, in order to address disparities in CSC involvement from the earliest stages of a child’s life.

In conclusion, this study provided novel data on CSC contact during pregnancy, demonstrating socio-economic and ethnic disparities between those in contact with CSC during pregnancy and those without. Adherence to pre-birth assessment guidance was only partially reflected in the study population, and this goes both ways: women with an indication to be referred, were not necessarily in contact with CSC; in contrast, a large proportion of women with CSC contact did not seem to have any identifiable indications for being referred. Consistent guidance, that provides clarity in thresholds while equally encouraging a holistic strengths-based family approach, is needed to address these inequalities, to reduce professional bias, and to ensure evidence-based practice is upheld nationally.

## Supplementary Information

Supplementary Material 1.

## Data Availability

The data used and/or analysed during the current study are available from the eLIXIR-Born in South London partnership. Data accessed remain within an NHS firewall and governance is provided by the eLIXIR Oversight Committee reporting to relevant information governance clinical leads. Subject to these conditions, data access is encouraged and those interested should contact the eLIXIR Chief Investigator (Professor Lucilla Poston; [Lucilla.poston@kcl.ac.uk)](mailto: Lucilla.poston@kcl.ac.uk)), https://www.kcl.ac.uk/research/elixir-1.

## Abbreviations

CSC: Children’s Social Care involvement
CRIS: Clinical Records Interactive Search
DA: Domestic Abuse
NHS: National Health Service
