# Correction: Contact with child protection services during pregnancy: a cross-sectional study using the eLIXIR born in South London, UK maternity-child data linkage

**DOI:** 10.1186/s12884-025-08476-1

**Published:** 2025-12-27

**Authors:** Kaat De Backer, Paul Seed, Sam Burton, Elsa Montgomery, Jane Sandall, eLIXIR-Born in South London Partnership, Abigail Easter

**Affiliations:** 1 Department of Women and Children’s Health, Faculty of Life Sciences and Medicine, King’s College London, London, UK; 2 School of Psychology, Liverpool John Moores University, Liverpool, UK; 3 Division of Methodologies, Florence Nightingale Faculty of Nursing, Midwifery and Palliative Care, King’s College London, London, UK

**Correction: BMC Pregnancy Childbirth 25**,** 1101 (2025)**

**https://doi.org/10.1186/s12884-025-08197-5**

Following publication of the original article [1], the authors identified an error in Fig. 4. The correct figure is given below. The original article has been corrected.

**Fig. 4:**
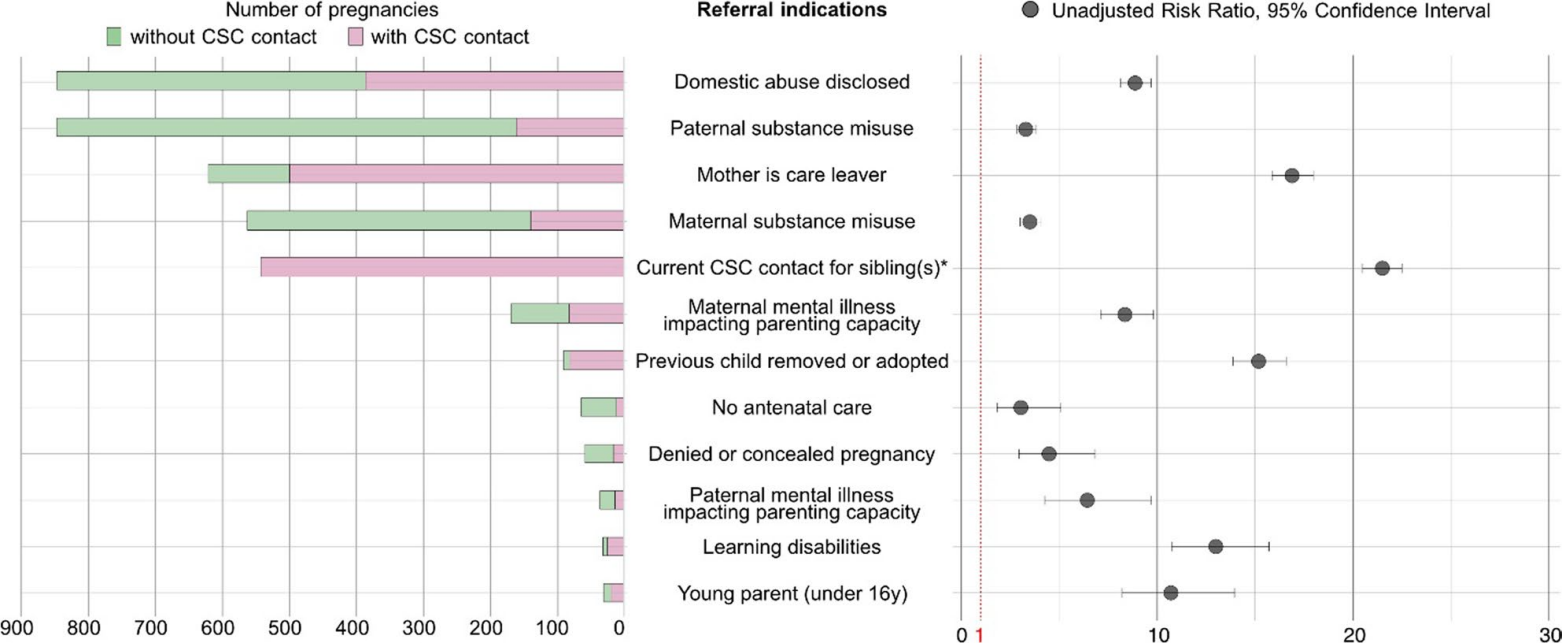
Referral indications and actual contact, in frequencies and unadjusted risk ratios
