# Supplementary material for: Contact with child protection services during pregnancy: a cross-sectional study using the eLIXIR Born in South London, UK maternity-child data linkage
Source: BMC Pregnancy Childbirth. 2025 Oct 16;25:1101. doi: 10.1186/s12884-025-08197-5 (PMC12532829; doi:10.1186/s12884-025-08197-5)
Supplement: Supplementary file 1 — Supplementary Material 1. [file 12884_2025_8197_MOESM1_ESM.docx]

**Supplementary materials**

| **Supplementary Table S1: Members of the Early Life Cross Linkage in Research, Born in South London (eLIXIR-BiSL) Partnership** | |
| --- | --- |
| **Member** | **Role** |
| Professor Lucilla Poston | eLIXIR-BiSL Chief Investigator, and Professor of Maternal & Fetal Health, Department of Women and Children’s Health, School of Life Course and Population Sciences, King’s College London |
| Professor Laura A Magee | Professor of Women’s Health, Department of Women and Children’s Health, School of Life Course and Population Sciences, King’s College London |
| Professor Robert Stewart | Professor of Psychiatric Epidemiology & Clinical Informatics, Department of Psychological Medicine, Institute of Psychiatry, Psychology and Neuroscience, King’s College London and NIHR Maudsley Biomedical Research Centre, South London and Maudsley NHS Foundation Trust, London. Consultant Psychiatrist at South London and Maudsley NHS Foundation Trust, London |
| Professor David Edwards | Chair in Paediatrics & Neonatal Medicine, Department of Perinatal Imaging and Health, King’s College London. Neonatal Consultant at Guy’s and St. Thomas’ NHS Foundation Trust |
| Professor Mark Ashworth | Professor of Primary Care, Department of Population Health Sciences, School of Life Course and Population Sciences, King’s College London |
| Professor Jane Sandall | Professor of Social Science & Women’s Health, Department of Women and Children’s Health, School of Life Course and Population Sciences, King’s College London |
| Dr Ingrid Wolfe | Professor of Paediatrics and Child Population Health, Department of Women and Children’s Health, School of Life Course and Population Sciences, King’s College London and Consultant in Children's Public Health Medicine and Director of the Evelina London Children’s Healthcare |
| Dr Cheryl Gillett | Head of Tissue Banking, Department of Comprehensive Cancer Centre, School of Cancer & Pharmaceutical Sciences, King’s College London |
| Dr Michael Absoud | Paediatric Consultant at Evelina London Children’s Healthcare |
| Dr Lucy Pickard | Consultant Paediatrician, King’s College Hospital NHS Foundation Trust |
| Ms Amanda Grey | Lay member of the eLIXIR Oversight Committee |
| Ms Sarah Spring | Lay member of the eLIXIR Oversight Committee |
| Ms Toyin Kazeem | Information Governance Operations Lead, South London and Maudsley NHS Foundation Trust, London |
| Ms Amelia Jewell | Clinical Data Linkage Service Lead, NIHR Maudsley Biomedical Research Centre, South London and Maudsley NHS Foundation Trust |
| Mr Matthew Broadbent | CRIS Clinical Informatics Lead, NIHR Maudsley Biomedical Research Centre, South London and Maudsley NHS Foundation Trust, London |
| Ms Finola Higgins | Research Informatics Programme Manager, Guy’s and St Thomas’ NHS Foundation Trust |
| Mr Leonardo de Jongh | Data Warehouse Manager, Guy’s and St. Thomas’s Hospital NHS Foundation Trust |
| Ms Tisha Dasgupta | Research Associate and eLIXIR Coordinator, Department of Women & Children’s Health, School of Life Course and Population Sciences, King’s College London |
| Dr Carolyn Gill | School Bioresource Manager, School of Life Course and Population Sciences, King’s College London. |

| **Supplementary Table S2: Pre-birth referral and assessment guidance and corresponding database variables** | | |
| --- | --- | --- |
| **Pre-birth referral and assessment guidance** | **Dataset^*^** | **How identified in database?** |
| A child has previously sustained non accidental injuries in the care of either parent / carer (this includes the sudden, unexpected death of a child where safeguarding concerns were raised); | Not able to retrieve | - |
| Previous children in the family have been removed from the care of the parent(s) either by a private arrangement or by a court order | DS8 Social Care dataset | Single variables: PreviousChildFosterCare; PreviousChildAdoption  Combined variables: PreviousChildLivingElsewhere AND PreviousSocialServicesInvolvement |
| A child in the household is the subject of a Child in Need or Child Protection Plan or is a Looked after Child | DS1 Booking Dataset & DS8 Social Care dataset | Parity>0 AND HasSocialServicesInvolvement at booking |
| Either parent is the subject of a Child in Need or Child Protection Plan or is a Looked after Child or Care Leaver | DS1 Booking dataset & DS8 Social Care dataset | Parity=0 AND HasSocialServicesInvolvement at booking  AccommodatedInChildhood |
| The mother is a child aged under 16 who is found to be pregnant | DS1 Booking dataset | MaternalAgeAtBooking equal or under 16y |
| A parent or other adult in the household, or regular visitor, has been identified as posing a risk to children | Not able to retrieve | - |
| There are concerns about the parent(s) ability to protect the baby | Not able to retrieve | - |
| There are concerns regarding domestic violence and abuse | DS7 Domestic Abuse dataset & CRIS | CurrentDomesticViolenceDiscolsed; CurrentDomesticAbuseReferralRequired; CurrentDomesticAbusePoliceInvolvement; CRIS_CurrentDA |
| Either or both parents have mental health problems that might impact on the care of a child | CRIS – Maternal Mental health | Any inpatient psychiatric admission during pregnancy; any detentions under the Mental Health Acti during pregnancy, any involvement from the following specialist teams during pregnancy: Home Treatment, Addictions, Forensic or Child and Adolescent Mental Health) |
|  | DS1 Booking dataset | MentalHealthProblemsPartner (free text search for any severe mental illness, including psychosis, psychotic*, schizophrenia) |
| Either or both parents have a learning disability that might impact on the care of a child | DS1 Booking Dataset, DS2 Intrapartum dataset | LearningDifficulities (free text search for any mentioning of learning disabilities, cognitive delay, low IQ,..) |
| Either or both parents abuse substances; alcohol or drugs | Maternal: DS1 Booking dataset & DS2 Intrapartum dataset, CRIS | Any disclosure during the pregnancy episode of alcohol or drug use (eg. CurrentRecreationalDrugsOrSubstancesAtBooking,  Any treatment under Addiction services during pregnancy |
|  | Paternal: DS1 Booking dataset | CurrentPartnerUsingDrugs |
| Any other concerns exist that the baby may be at risk of Significant Harm including a parent previously suspected of fabricating or inducing illness in a child or harming a child | Not able to retrieve | - |
| The mother had not registered for antenatal care | DS1 Booking dataset & DS2 Intrapartum dataset | SocialRiskFactors identified at booking or during intrapartum care (NoAntenatalCare) |
| The pregnancy is denied or concealed | DS1 Booking dataset | LateBookingReason: “Concealed pregnancy” LateBookingReason: “Undecided about continuing pregnancy” AND GestationAtBooking >24wks |

Badgernet datasets are grouped according to timepoint, nature of the variables and clinical area where data was recorded: DS1 Booking dataset, DS2 Intrapartum dataset, DS7 Domestic abuse enquiry dataset, DS8 Social Risk factors enquiry dataset

| **Supplementary Table S3: Number of risk factors identified per pregnancy episode and association with CSC involvement** | | | | |
| --- | --- | --- | --- | --- |
| **Number of risk factors identified** | **Total number of pregnancies n (%)** | **Number of pregnancies with CSC contact n (%)** | **RR (95%CI)** | **p-value** |
| **0** | 33,296 (91.67%) | 913 (2.74%) | Reference | - |
| **1** | 2,404 (6.62%) | 841 (34.98%) | 12.76 (11.72-13.89) | <0.0001 |
| **2** | 438 (1.21%) | 284 (64.84%) | 23.65 (21.50-26.00) | <0.0001 |
| **3** | 124 (0.34%) | 111 (89.52%) | 32.65 (29.87 -35.68) | <0.0001 |
| **4** | 49 (0.13%) | 46 (93.88%) | 34.24 (31.07-37.72) | <0.0001 |
| **5** | 11 (0.03%) | 11 (100%) | 1 | - |
